# Supplementary material for: Inferring chromosome radial organization from Hi-C data
Source: BMC Bioinformatics. 2020 Nov 10;21:511. doi: 10.1186/s12859-020-03841-7 (PMC7654587; doi:10.1186/s12859-020-03841-7)
Supplement: Supplementary file 1 — Additional file 1. This file contains Tables S1−S2 and Figures S1−S11. [file 12859_2020_3841_MOESM1_ESM.pdf]

Supplementary Materials for

Inferring Chromosome Radial Organization from Hi-C Data

Priyojit Das[1], Tongye Shen[2], and Rachel Patton McCord[2,*]

[1]UT-ORNL Graduate School of Genome Science and Technology, University of Tennessee, Knoxville, TN 37996, USA
[2]Department of Biochemistry & Cellular and Molecular Biology, University of Tennessee, Knoxville, TN 37996, USA

[*]Correspondence to: rmccord@utk.edu

This file includes:
Table S1 and S2
Figs S1 to S11

**Cell Line**	**Type**	**Nuclear Shape**	**Hi-C Data**	**GEO or EGA Accession Number**	**Microscopy Data**
GM12878	Lymphoblastoid	Spherical	Sanders et al. (2019)	GSE136899	Boyle et al. (2001)
GM12878 Rao	Lymphoblastoid	Spherical	Rao et al. (2014)	GSE63525	Boyle et al. (2001)
BJ1-hTERT	Skin Fibroblast	Ellipsodial	Sanders et al. (2019)	GSE136899	Bolzer et al. (2005)
BJ-5ta	Skin Fibroblast	Ellipsoidal	Sanders et al. (2019)	GSE136899	Bolzer et al. (2005)
MCF10A	Breast Epithelium	Ellipsoidal	Barutcu et al. (2015)	GSE66733	Fritz et al. (2014)
HMEC	Breast Epithelium	Spherical	Rao et al. (2014)	GSE63525	-
Neutrophil	Neutrophil	Multi Lobed	Javierre et al. (2016)	EGAS00001000327	-
WI38-hTERT	Lung Fibroblast	Ellipsoidal	Chandra et al. (2015)	ENA PRJEB8073	-
WI38-hTERT Senescent	Lung Fibroblast	Ellipsoidal	Chandra et al. (2015)	ENA PRJEB8073	-
HGPS Patient p19	Lymphoblastoid	Ellipsoidal	McCord et al. (2013)	GSE41763	-

Supplementary Table 1: Dataset description

	Correlation with microscopy data from:		
	GM12878	BJ1-hTERT	MCF10A
Pairwise strong contact PC1 for given cell type	-0.873	-0.791	0.763
Gene Density	-0.8	-0.3	0.312
Chromosome Length	0.43	0.89	0.959
Tuned network model for given cell type	0.811	0.848	0.941

Supplementary Table 2: Correlation between microscopy data radial CT positions and those inferred by different sources of information

Supplementary Figure 1 Radial CT arrangement of GM12878 (FitHiC). **a)** Pairwise inter-chromosomal significant interaction pattern matrix derived from FitHiC for GM12878 Hi-C data. **b)** 2D PCA projection of the FitHiC pairwise inter-chromosomal significant interaction pattern matrix obtained from GM12878 Hi-C data. **c)** Network modeling generated model cluster for FitHiC GM12878 contacts, selected based on inferred CT distribution type. **d)** Correlation between predicted tuned CT distance from GM12878 (FitHiC) and lymphoblastoid microscopy imaging data. **f)** Correlation between predicted tuned CT distances obtained from GM12878 (FitHiC) and GM12878 (strong interactions)

Supplementary Figure 2 Radial CT arrangements inferred from GM12878 and BJ1-hTERT simulated random ligation data. **a)** Network modeling cluster for GM12878 simulated random ligation, selected based on inferred CT distribution type. **b)** Correlation between predicted tuned CT distance from GM12878 simulated random ligation Hi-C data and lymphoblastoid microscopy imaging data. **c)** Pairwise inter-chromosomal strong interaction pattern matrix for BJ1-hTERT simulated random ligation Hi-C data. **d)** 2D PCA projection of the pairwise inter-chromosomal strong interaction pattern matrix obtained from BJ1-hTERT simulated random ligation Hi-C data. **e)** Network modeling cluster for BJ1-hTERT simulated random ligation, selected based on inferred CT distribution type. **f)** Correlation between predicted tuned CT distance from BJ1-hTERT simulated random ligation Hi-C data and fibroblast microscopy imaging data.

Supplementary Figure 3 Network modeling generated clusters for GM12878. For each cluster, the Pearson's correlation of mean radial CT distances with gene density (GD) and chromosome length (LN) are shown on the top of the cluster. Number of models in each cluster is indicated in parentheses above each cluster.

Supplementary Figure 4 The radial distance distributions of 23 CTs for both GM12878 and BJ1-hTERT obtained from the respective selected model clusters based on inferred CT distribution types (# of models in the selected clusters- GM12878: 109, and BJ1-hTERT: 122). All distributions are ordered left to right from center to periphery.

Supplementary Figure 5 Comparison of network modeling generated radial CT distance profiles with experimentally obtained distributions. **a-b)** The radial distance profiles of CT18 and CT19 **(a top)** and short and long chromosomes **(b top)** for GM12878 and BJ1-hTERT obtained from the network model clusters in this current work. These distributions are similar to the radial arrangement previously measured by microscopy for CT18 and CT19 **(a bottom)** and short and long chromosomes **(b bottom)** in lymphocyte (2D arrangement) and fibroblast (3D arrangement) nuclei. Panel **(a-b)** bottom parts are adapted from Cremer et al. (2001). Copyright Springer Nature. Used with permission. **c) Top** - The radial

distance profiles of CT10 and CTX for BJ1-hTERT obtained from our network model cluster. **Bottom** - The distance distribution of CT10 and CTX from the nuclear periphery in human dermal firbroblasts obtained using 3D FISH. Panel **(c)** bottom part is created with data obtained from Mehta et al. (2010). **d) Top** - The radial distance profiles of CT4 for GM12878 obtained from our network modeling cluster. **Bottom** - Radial distance profiles of AF4 gene, which is located on chr4, from the nucleus center in two lymphoblastic cell lines - NALM-6 (black) and IL-9 (grey). Panel **(d)** bottom part is adapted from Gué et al. (2005), copyright John Wiley and Sons, used with permission.

Supplementary Figure 6 The radial distance distribution profiles of 23 CTs for GM12878, GM12878 simulated random ligation, BJ1-hTERT and BJ1-hTERT simulated random ligation obtained from the respective selected model clusters based on inferred CT distribution types (# of models in the selected clusters: for GM12878 - 109, GM12878 simulated random ligation - 95, BJ1-hTERT - 122, and BJ1-hTERT simulated random ligation - 113).

Supplementary Figure 7 Radial arrangement of BJ1-hTERT and BJ-5ta follow similar CT distribution patterns. **a)** Pairwise inter-chromosomal strong interaction pattern matrix for BJ-5ta Hi-C data. **b)** 2D PCA projection of the pairwise inter-chromosomal strong interaction pattern matrix obtained from BJ-5ta Hi-C data. **c)** Network modeling generated model cluster for BJ-5ta, selected based on respective inferred CT distribution type (# of models in the selected clusters: 115). **d)** Correlation of BJ1-hTERT PC1 values with BJ-5ta PC1 values obtained from the PCA transformation of the respective pairwise inter-chromosomal strong interaction pattern matrices. **e)** Correlation between predicted tuned CT distance from BJ-5ta Hi-C data and fibroblast microscopy imaging data. **f)** Correlation between predicted tuned CT distances obtained from BJ1-hTERT and BJ-5ta.

Supplementary Figure 8 Radial arrangement of GM12878 replicates R1 and R2 show similar CT distribution pattern. **a)** Pairwise inter-chromosomal strong interaction pattern matrix for GM12878 R1 Hi-C data (R2 used in main figures). **b)** 2D PCA projection of the pairwise inter-chromosomal strong interaction pattern matrix obtained from GM12878 R1 Hi-C data. **c)** Network modeling generated model cluster for GM12878 R1, selected based on respective inferred CT distribution type (# of models in the selected clusters: 142). **d)** Correlation of GM12878 R2 PC1 values with GM12878 R1 PC1 values obtained from the PCA transformation of the respective pairwise inter-chromosomal strong interaction pattern matrices. **e)** Correlation between predicted tuned CT distance from GM12878 R1 Hi-C data and lymphoblastoid microscopy imaging data. **f)** Correlation between predicted tuned CT distances obtained from GM12878 R2 and GM12878 R1

Supplementary Figure 9 The radial distance profiles of 23 CTs for GM12878 R1, GM12878 R2, BJ1-hTERT and BJ-5ta obtained from the respective selected model clusters based on inferred CT distribution types (# of models in the selected clusters: for GM12878 R2 - 109, GM12878 R1 - 142, BJ1-hTERT - 122, and BJ-5ta - 115)

Supplementary Figure 10 The radial distance profiles of 23 CTs for GM12878 standard, paternal, and maternal copies obtained from the respective selected model clusters based on inferred CT distribution types (# of models in the selected clusters: for GM12878 standard - 109, paternal copy - 194, and maternal copy - 148)

Supplementary Figure 11 Statistical comparisons of the CT distance profiles obtained from network modeling for GM12878 and BJ1-hTERT for different sample sizes n_s (number of network structures generated from Hi-C data) using the two-sided Mann-Whitney U test. The color values and the asterisk marks represent the level of significance of the BJ1-hTERT vs. GM12878 distribution difference. Green - P-value $\leq$ 0.0001 - ****; Blue - P-value $\leq$ 0.001 - ***; Yellow - P-value $\leq$ 0.01 - **; Red - P-value $\leq$ 0.05 - *; White - P-value $>$ 0.05 - non significant (ns)

Supplementary Figure 1

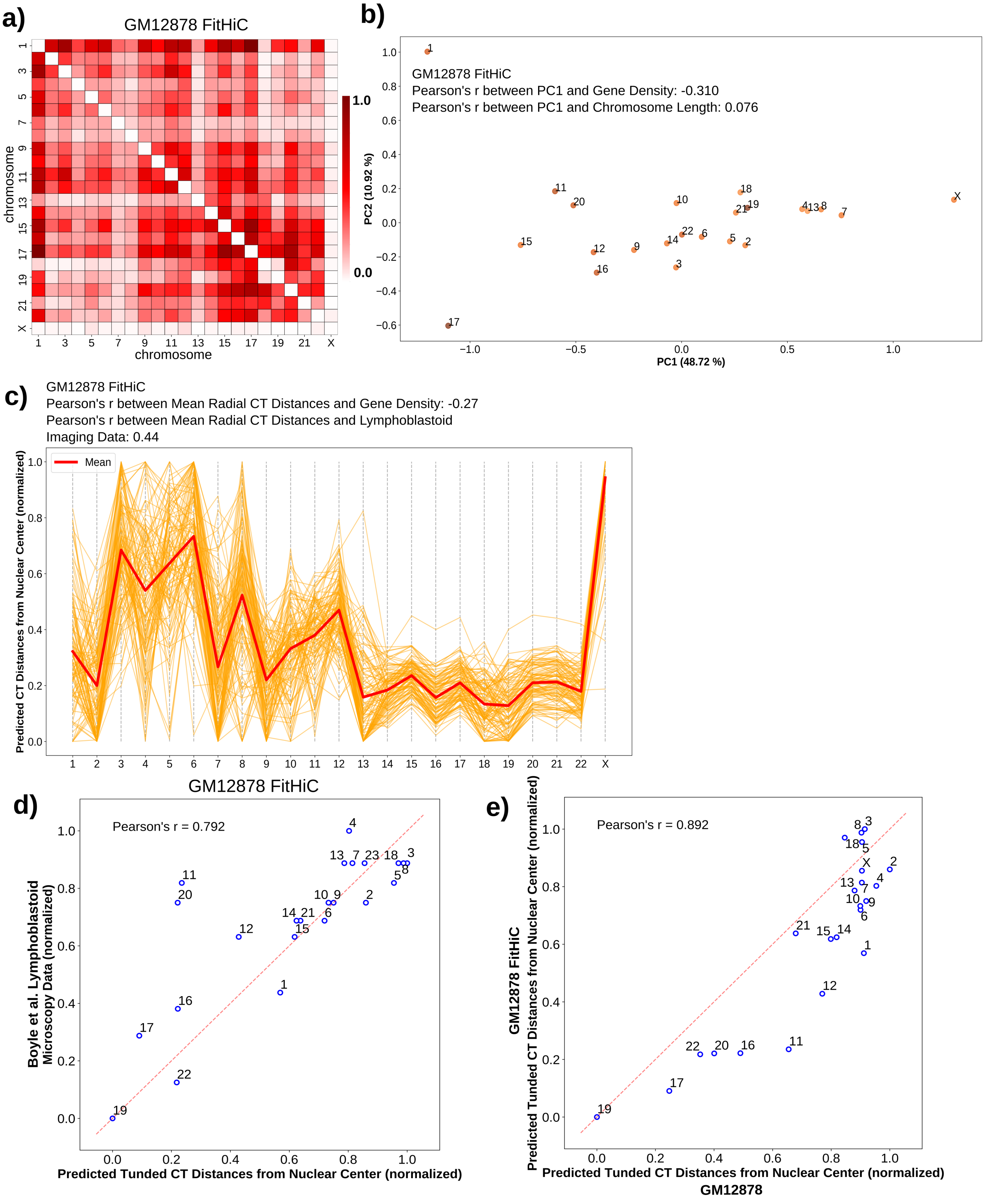

Supplementary Figure 2

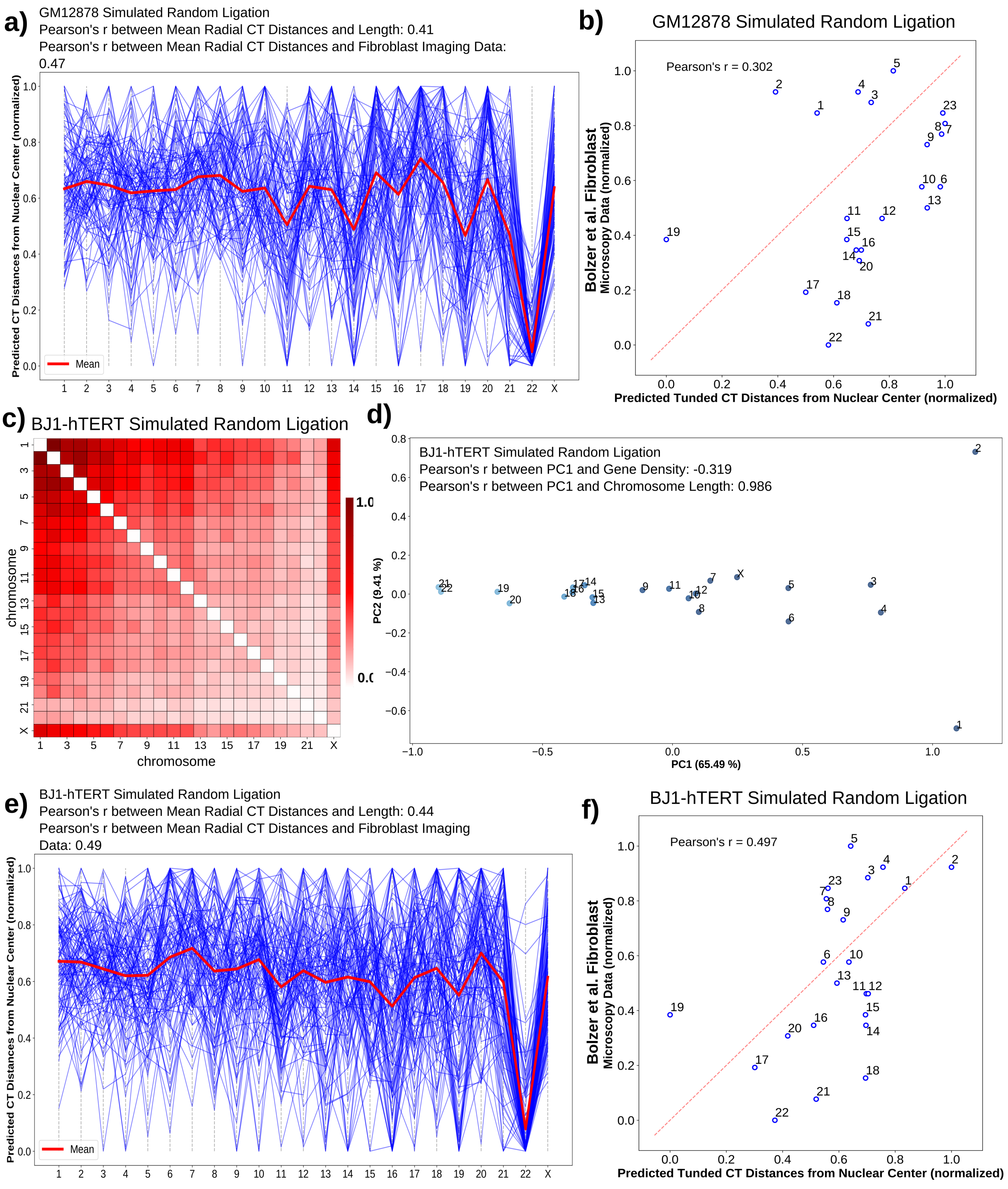

Supplementary Figure 3

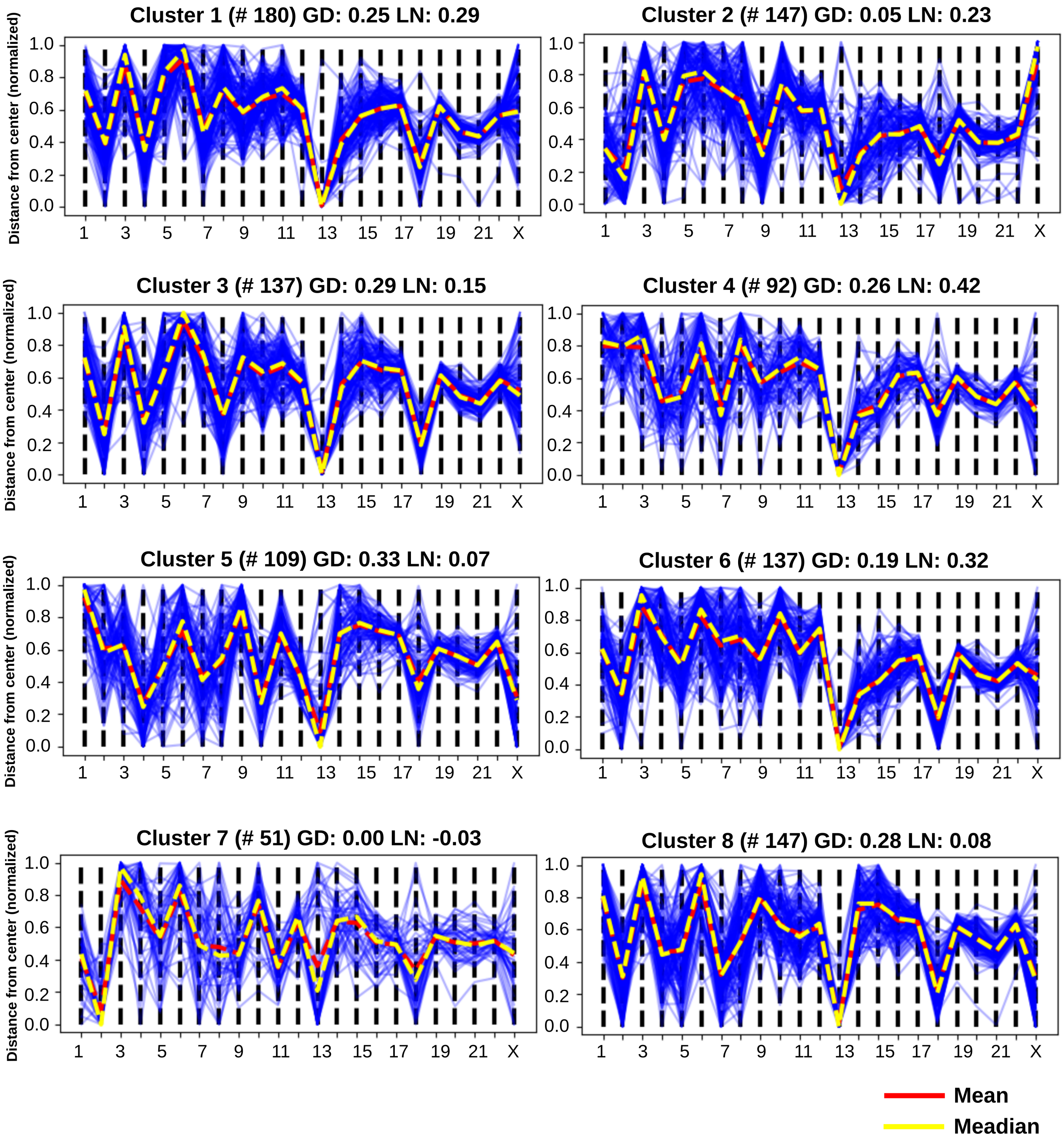

Supplementary Figure 4

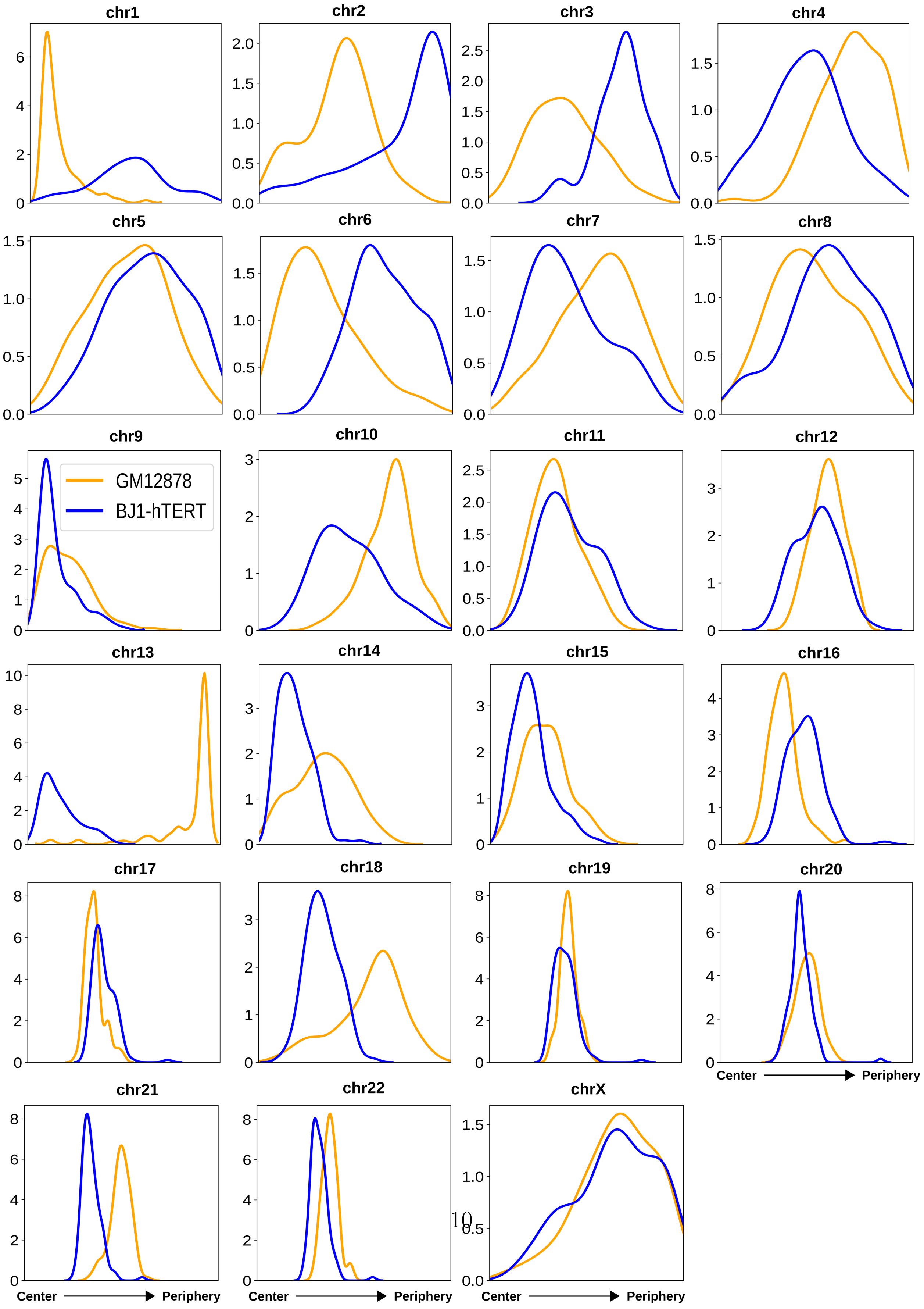

Supplementary Figure 5

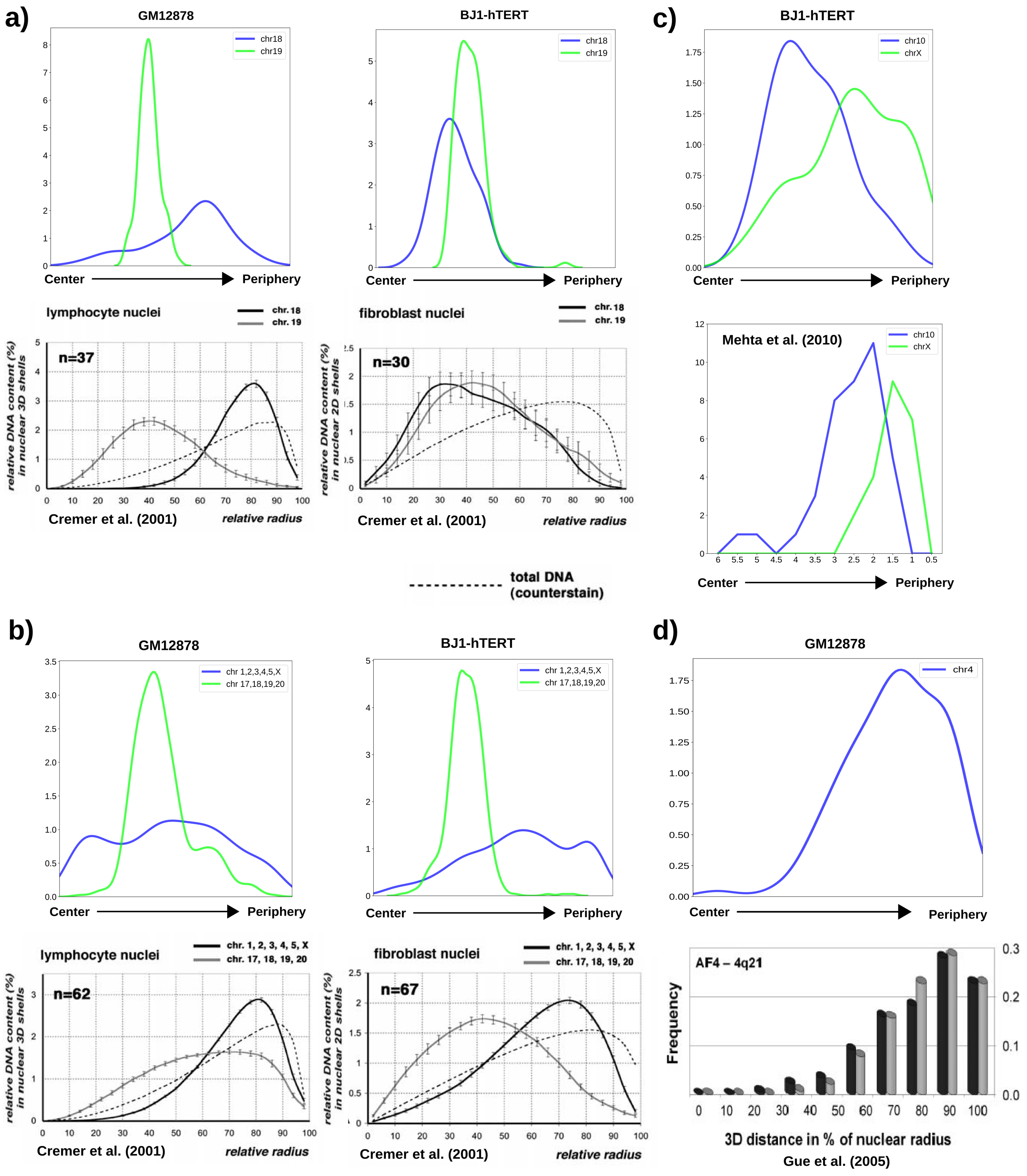

Supplementary Figure 6

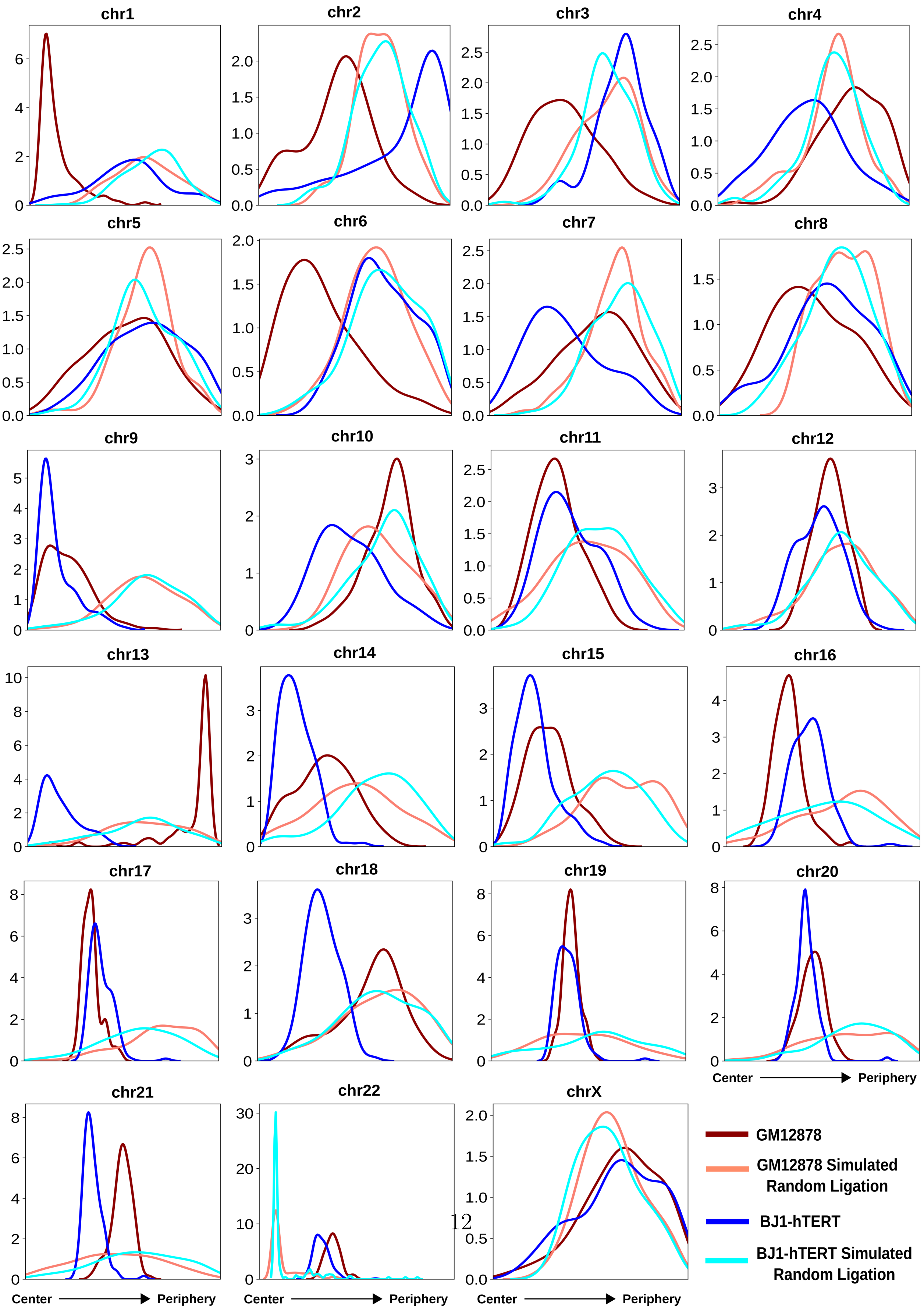

Supplementary Figure 7

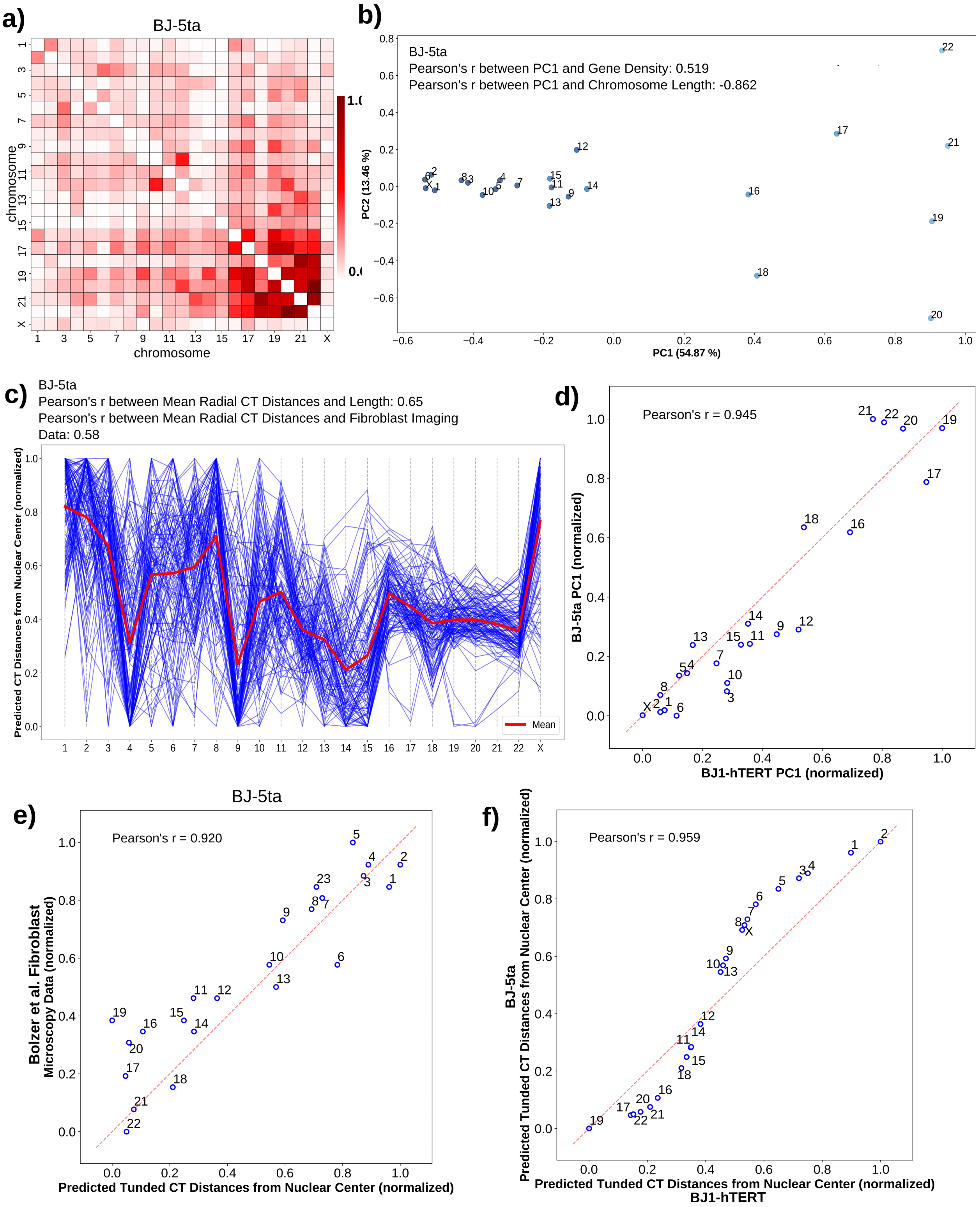

Supplementary Figure 8

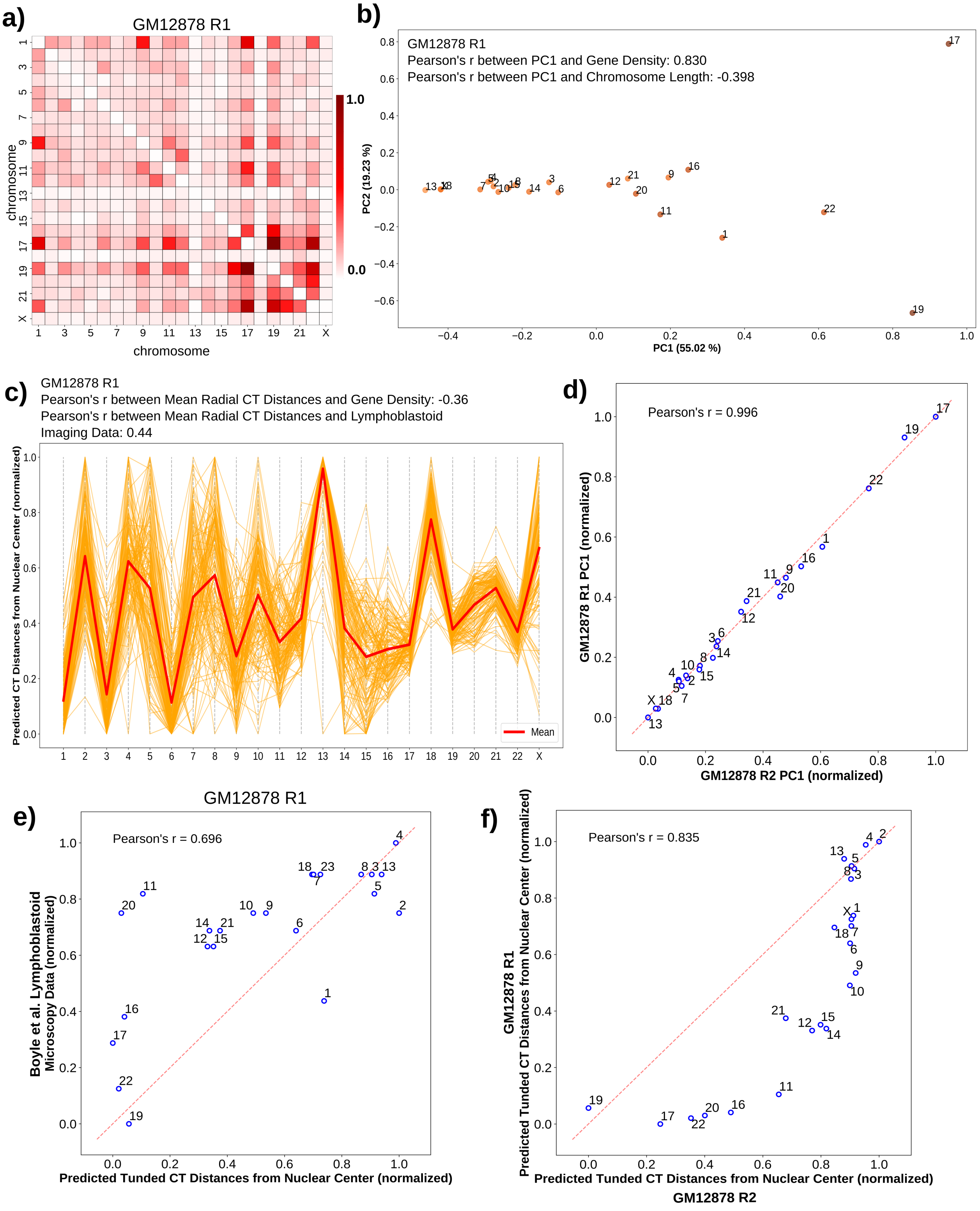

Supplementary Figure 9

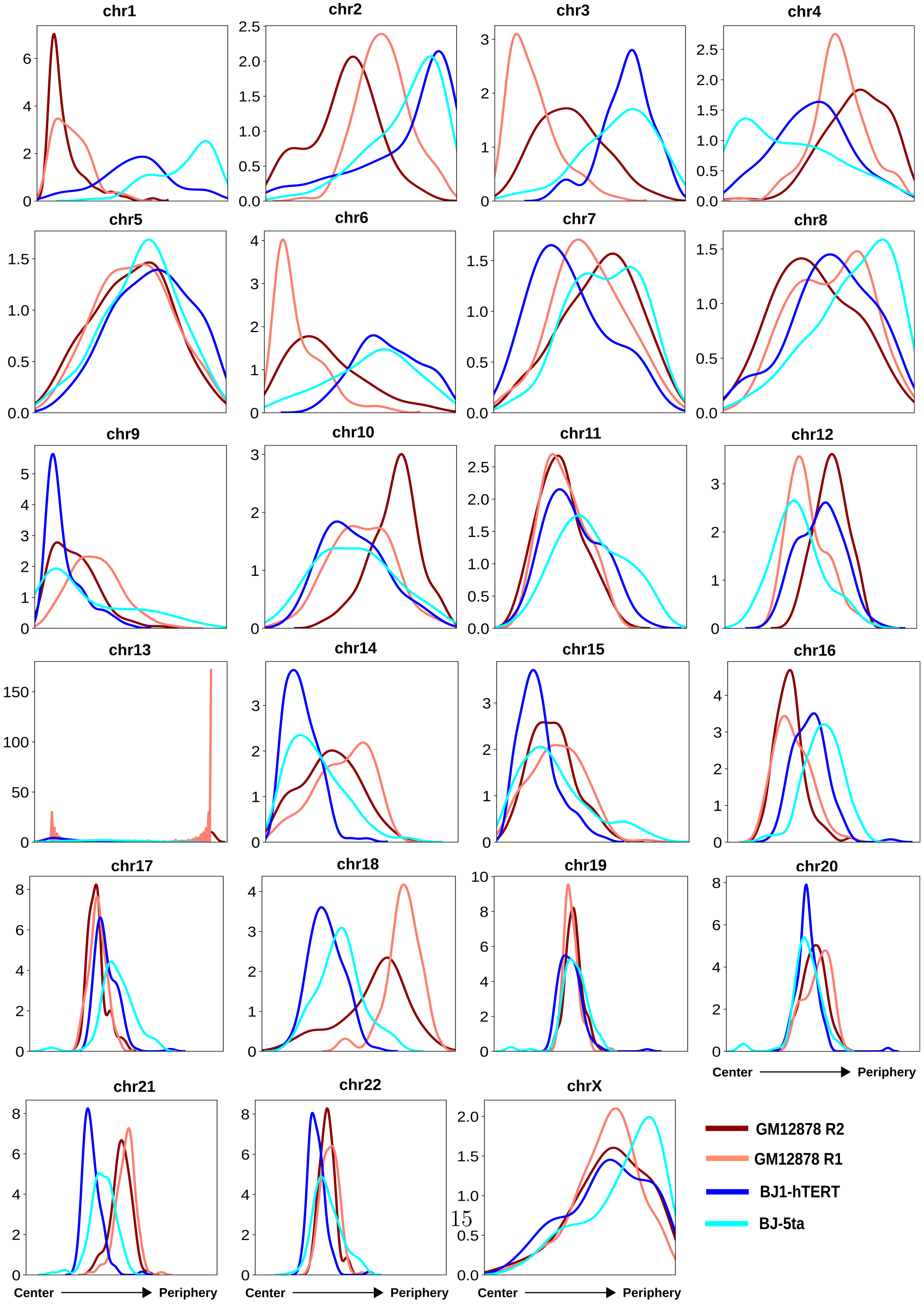

Supplementary Figure 10

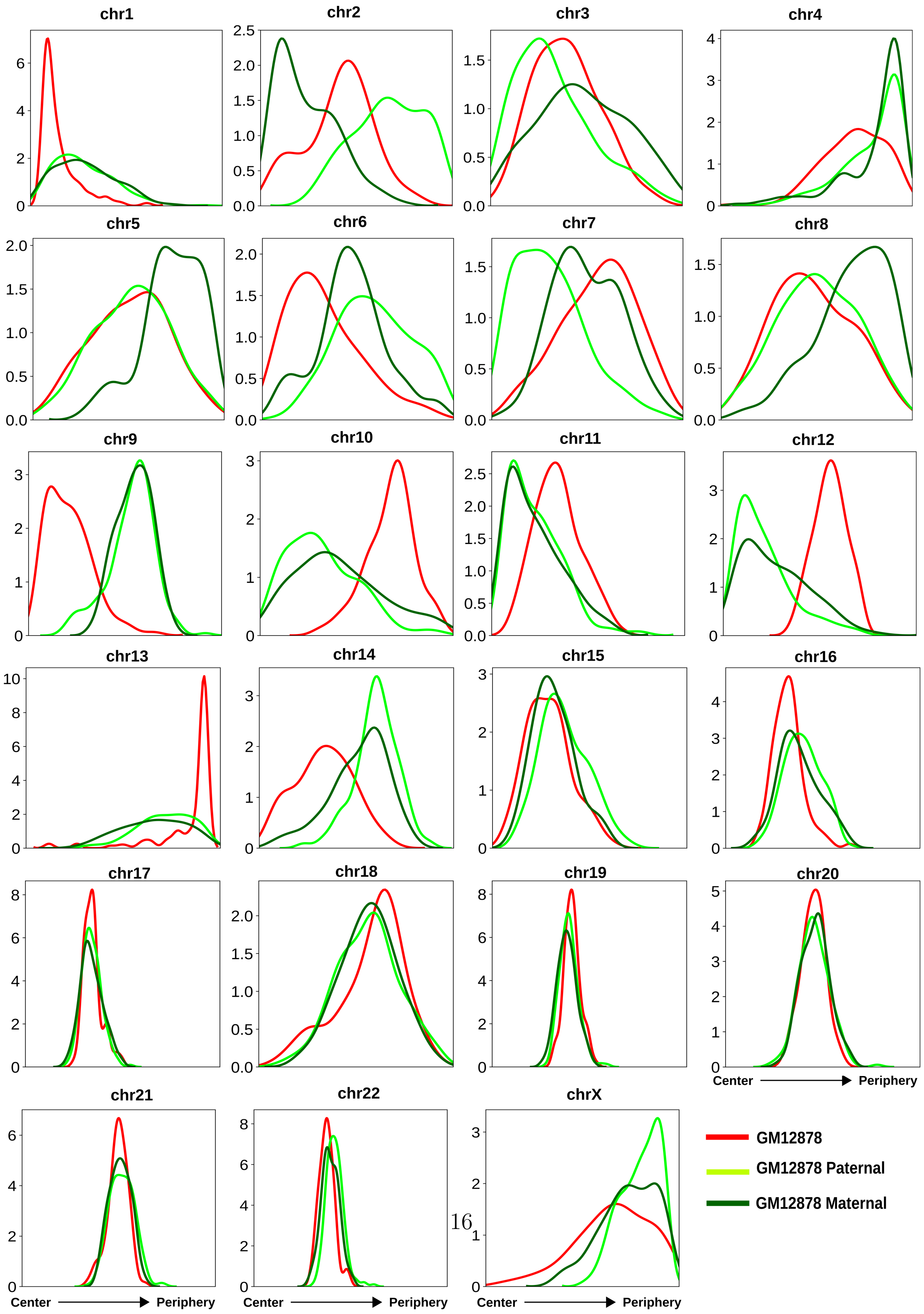

Supplementary Figure 11

chr	n_s = 100	n_s = 250	n_s = 500	n_s = 750	n_s = 1000
1	****	****	****	****	****
2	****	****	****	****	****
3	****	****	****	****	****
4	ns	**	****	****	****
5	ns	****	***	****	****
6	****	****	****	****	****
7	*	****	****	****	****
8	ns	ns	ns	ns	ns
9	**	****	****	****	****
10	ns	ns	**	****	****
11	ns	ns	ns	*	**
12	ns	ns	ns	*	ns
13	****	****	****	****	****
14	****	****	****	****	****
15	***	****	****	****	****
16	****	****	****	****	****
17	**	***	****	****	****
18	****	****	****	****	****
19	ns	*	***	****	****
20	**	****	****	****	****
21	****	****	****	****	****
22	*	****	****	****	****
X	*	**	***	****	****

References

Barutcu, A. R., Lajoie, B. R., McCord, R. P., Tye, C. E., Hong, D., Messier, T. L., Browne, G., van Wijnen, A. J., Lian, J. B., Stein, J. L., et al. (2015). Chromatin interaction analysis reveals changes in small chromosome and telomere clustering between epithelial and breast cancer cells. *Genome biology*, 16(1):214.

Bolzer, A., Kreth, G., Solovei, I., Koehler, D., Saracoglu, K., Fauth, C., Müller, S., Eils, R., Cremer, C., Speicher, M. R., et al. (2005). Three-dimensional maps of all chromosomes in human male fibroblast nuclei and prometaphase rosettes. *PLoS biology*, 3(5):e157.

Boyle, S., Gilchrist, S., Bridger, J. M., Mahy, N. L., Ellis, J. A., and Bickmore, W. A. (2001). The spatial organization of human chromosomes within the nuclei of normal and emerin-mutant cells. *Human molecular genetics*, 10(3):211–220.

Chandra, T., Ewels, P. A., Schoenfelder, S., Furlan-Magaril, M., Wingett, S. W., Kirschner, K., Thuret, J.-Y., Andrews, S., Fraser, P., and Reik, W. (2015). Global reorganization of the nuclear landscape in senescent cells. *Cell reports*, 10(4):471–483.

Cremer, M., Von Hase, J., Volm, T., Brero, A., Kreth, G., Walter, J., Fischer, C., Solovei, I., Cremer, C., and Cremer, T. (2001). Non-random radial higher-order chromatin arrangements in nuclei of diploid human cells. *Chromosome research*, 9(7):541–567.

Fritz, A. J., Stojkovic, B., Ding, H., Xu, J., Bhattacharya, S., Gaile, D., and Berezney, R. (2014). Wide-scale alterations in interchromosomal organization in breast cancer cells: defining a network of interacting chromosomes. *Human molecular genetics*, 23(19):5133–5146.

Gué, M., Messaoudi, C., Sun, J. S., and Boudier, T. (2005). Smart 3d-fish: automation of distance analysis in nuclei of interphase cells by image processing. *Cytometry Part A: The Journal of the International Society for Analytical Cytology*, 67(1):18–26.

Javierre, B. M., Burren, O. S., Wilder, S. P., Kreuzhuber, R., Hill, S. M., Sewitz, S., Cairns, J., Wingett, S. W., Várnai, C., Thiecke, M. J., et al. (2016). Lineage-specific genome architecture links enhancers and non-coding disease variants to target gene promoters. *Cell*, 167(5):1369–1384.

McCord, R. P., Nazario-Toole, A., Zhang, H., Chines, P. S., Zhan, Y., Erdos, M. R., Collins, F. S., Dekker, J., and Cao, K. (2013). Correlated alterations in genome organization, histone methylation, and dna–lamin a/c interactions in hutchinson-gilford progeria syndrome. *Genome research*, 23(2):260–269.

Mehta, I. S., Amira, M., Harvey, A. J., and Bridger, J. M. (2010). Rapid chromosome territory relocation by nuclear motor activity in response to serum removal in primary human fibroblasts. *Genome biology*, 11(1):1–17.

Rao, S. S., Huntley, M. H., Durand, N. C., Stamenova, E. K., Bochkov, I. D., Robinson, J. T., Sanborn, A. L., Machol, I., Omer, A. D., Lander, E. S., et al. (2014). A 3d map of the human genome at kilobase resolution reveals principles of chromatin looping. *Cell*, 159(7):1665–1680.

Sanders, J. T., Freeman, T. F., Xu, Y., Golloshi, R., Stallard, M. A., Martin, R. S., Balajee, A. S., and McCord, R. P. (2019). Radiation-induced dna damage and repair effects on 3d genome organization. *bioRxiv*.
